# Jefferson Medical College Commencement

**Published:** 1830

**Authors:** 


31. At the late public commencement in the Medical Department of Jefferson College, the degree of Doctor of Medicine was conferred on thirty-five young gentlemen, alumni of the institution.

